# Characteristics and Health Risks of Phthalate Ester Contamination in Soil and Plants in Coastal Areas of South China

**DOI:** 10.3390/ijerph19159516

**Published:** 2022-08-03

**Authors:** Huanhuan Xing, Xiaolong Yu, Jiahui Huang, Xiaodong Du, Mengting Wang, Jianteng Sun, Guining Lu, Xueqin Tao

**Affiliations:** 1 Key Laboratory of Ministry of Education on Pollution Control and Ecosystem Restoration in Industry Clusters, Guangdong Provincial Engineering and Technology Research Center for Environmental Risk Prevention and Emergency Disposal, School of Environment and Energy, South China University of Technology, Guangzhou 510006, China; ncuspyxinghuanhuan@163.com (H.X.); 13580436478@163.com (X.D.); wangmengting823@163.com (M.W.); 2 Guangdong Provincial Key Laboratory of Petrochemical Pollution Processes and Control, School of Environmental Science and Engineering, Guangdong University of Petrochemical Technology, Maoming 525000, China; yzxendeavour@163.com (X.Y.); hjh960409@163.com (J.H.); 3 College of Resources and Environment, Zhongkai University of Agriculture and Engineering, Guangzhou 510225, China; xqtao@foxmail.com

**Keywords:** bis(2-ethylhexyl) phthalate, di-n-butyl phthalate, noncarcinogenic risk, carcinogenic risk, agriculture soil, leafy vegetables

## Abstract

Phthalate esters (PAEs) are widely used as plasticizers in industrial and commercial products, and are classified as endocrine-disrupting compounds. In this study, we investigated the contamination characteristics and health risks of PAEs in the soil–plant system in coastal areas of South China. PAEs were detected in soil and plant samples at all 37 sampling sites. The total concentration of the 15 PAEs in soil samples ranged from 0.445 to 4.437 mg/kg, and the mean concentration was 1.582 ± 0.937 mg/kg. The total concentration of the 15 PAEs in plant samples ranged from 2.176 to 30.276 mg/kg, and the mean concentration was 8.712 ± 5.840 mg/kg. Di(2-Ethylhexyl) phthalate (DEHP) and di-n-butyl phthalate (DnBP) were the major PAEs compounds in all samples. The selected contaminants exhibited completely different spatial distributions within the study area. Notably, higher concentrations of PAEs were found in the coastal Guangdong Province of South China. The average noncarcinogenic risks of Σ6 PAEs were at acceptable levels via dietary and nondietary routes. However, the noncarcinogenic risks posed by DEHP and DBP at some sampling sites were relatively high. Furthermore, dietary and nondietary carcinogenic risks were very low for BBP, but carcinogenic risks posed by DEHP via diet. The results suggest that PAEs in the coastal soil–plant system in South China, through human risk assessment, will induce some adverse effects on human health, especially in children. This study provides an important basis for risk management of PAEs in agriculture, and safety in coastal areas of South China.

## 1. Introduction

Since the 1970s, the study of phthalate esters (PAEs) in the environment has attracted a lot of attention in China and abroad. PAEs are among the most abundant and widely used synthetic organic compounds, and are commonly used as additives in plastics, food packaging, personal care products, pesticides, and agricultural films [1]. In the plastic industry, they are mainly used as plasticizers and softeners to increase the flexibility of plastics. PAEs are noncovalently bound to plastic products, rather than chemically bound, and exist in a free and leachable phase [2]. Therefore, they are gradually and continuously released into the environment, causing widespread contamination of the atmosphere [3], water [4], soil [5,6,7], sediment [8,9], plants [10,11], and food [12]. PAEs have been detected in a variety of environmental matrices, resulting in a significant potential hazard to the environment and public health.

Soil is a major reservoir of hydrophobic organic pollutants, including PAEs [13]. In recent years, the soil of China has been heavily contaminated by frequent agricultural practices, industrial production, urban development, and human activities [14]. PAEs can be released into the soil from a variety of sources, including agricultural irrigation, agricultural plastic mulch, solid waste disposal, municipal solid organisms, and some other off-site sources of pollution [15]. Therefore, PAE contamination is still a serious problem in China based on recent data from PAE contamination in soils [16,17]. Once PAEs enter the soil matrix, they can be absorbed by plants, which in turn can cause damage to vegetables, and be passed through the food chain to humans [18,19]. Dietary intake is considered to be one of the important routes of intake of PAEs in humans [20], with plant-based food intake making up the bulk of the diet [21]. As a consequence, with the increased attention and concerns about the quality and safety of plant foods, several studies have focused on the uptake and bioaccumulation of PAEs in plants, mainly in leafy vegetables [11,22,23]. It was found that PAEs can be metabolized and concentrated in plants by uptake from the soil [22,24]. The contamination of PAEs in soil–plant systems has received increasing attention, and some studies had revealed the characteristics and health risks of PAEs in soil–plant systems in the Yangtze River Delta [25], the Dalian region of Northeast China, and agricultural plastic-film-covered land [26,27]. These studies are important for providing countermeasures for pollutant control.

In addition, high concentrations of PAEs in soils and plants pose a potential threat to human health. PAEs are highly fat-soluble, and can enter humans and animals through food intake, breathing contaminated air, drinking water, skin absorption, etc. [28]. As lipophilic chemicals, PAEs are molecularly similar to endogenous estrogens, and they are suspected to be endocrine disruptors (EDCs) [29]. PAEs have potential carcinogenic, teratogenic, and mutagenic effects [30]. PAEs and their metabolites can erroneously interact with molecular endocrine signaling systems [31], acting as hormone sensitizers by disrupting or impairing normal physiological mechanisms. This would be directly reflected in the dysfunction of body systems, leading to adverse health effects [30]. As toxicity studies progress, PAEs have been proven to pose a threat to children and adolescents, including epigenetic regulation, male and female reproduction, insulin resistance, type II diabetes, overweight, obesity, bone abnormalities, allergies, asthma, and even cancer [32]. Therefore, the negative impacts of PAEs on human health and biota are of great concern. Many studies have assessed the health risks of PAEs in plants and soils, including noncarcinogenic risk assessment of bis(2-ethylhexyl) phthalate (DEHP), butyl benzyl phthalate (BBP), di-n-butyl phthalate (DnBP), dimethyl phthalate (DMP), diethyl phthalate (DEP), and di-n-octyl phthalate (DnOP) under dietary and nondietary routes, and carcinogenic risk assessment of DEHP and BBP under dietary and nondietary routes [25,26].

The coastal region of South China is a popular tourist destination. The region includes the coastlines of three provinces (Guangxi, Guangdong, and Fujian), with the Pearl River Delta on the Guangdong coastline being one of the most populous and economically prosperous regions in the world [33], due to the rapid development of coastal cities and the demand for agricultural products, such as vegetables, in urban areas. A large number of agricultural activities are carried out, and a large number of industrial enterprises are concentrated. Due to their geographical location, many aquaculture and processing plants are located in the coastal areas of South China [34]. PAEs may be present at high concentrations in soils and plants in coastal areas of South China. However, few studies have characterized PAEs, and studies on PAE contamination in China have generally focused on relatively small sampling areas [5,27,35], Despite the fact that PAEs are one of the typical plasticizers in coastal areas of South China. Thus, to portray the environmental distribution and characteristics of PAEs in soil–plant systems in the coastal areas of South China. We planned a coastal sampling route consisting of 37 sites covering about 13,000 km of coastline in the coastal cities of South China, covering three provinces, and sampled soil and plants at each site.

The objectives of this study were: (I) to investigate the concentrations and distribution of 15 PAEs in soils and their corresponding plants in coastal areas of southern China; (II) to characterize the uptake of PAEs in soil–plant systems and their contamination; and (III) to assess the carcinogenic and noncarcinogenic risks of PAEs on the local population (adults and children) through dietary and nondietary routes, and the estrogenic effects of PAEs on humans.

## 2. Materials and Methods

### 2.1. Sampling Information

A total of 37 topsoil and 37 plant samples were collected from agricultural regions of 20 cities in Guangdong Province, Guangxi Province, and Fujian Province in the coastal region of South China. Guangdong Province included Chaozhou City (CZ), Maoming City (MM), Yangjiang City (YJ), Zhanjiang City (ZJ), Shanwei City (SW), Shantou City (ST), Jieyang City (JY), Dongguan City (DG), Jiangmen City (JM), Zhuhai City (ZH), Zhongshan City (ZS), Huizhou City (HZ), Guangzhou City (GZ), Shenzhen City (SZ); Guangxi Province included Fangchenggang City (FCG), Beihai City (BH), and Qinzhou City (GXQZ); and Fujian Province included Quanzhou City (QZ), Xiamen City (XM), and Zhangzhou City (ZZ). All 37 sampling sites are presented in Table S1. Soil and plant sampling was conducted in July 2019. To ensure the representativeness of the samples collected, the topsoil was collected from 0–10 cm using the S-shape method, and the plant samples were collected from leafy vegetables in the vicinity of the soil samples. The latitude and longitude of the sampling sites were recorded using GPS. All samples were placed in sealed polyethylene kraft paper bags, and sent immediately to the laboratory. Soil and plant samples were stored at −40 °C. Soil samples were freeze-dried to remove stones, plant tissue, and other debris, and subsequently ground and passed through a 1 mm sieve. Plant samples were freeze-dried and ground through a 0.15 mm sieve. Both the mortar and sieve were washed with deionized water and dried before use to avoid contamination.

### 2.2. Chemicals and Reagents

A standard mixture of 15 PAEs consisting of dibutyl phthalate (DnBP), bis(2-ethylhexyl) phthalate (DEHP), di-n-octyl phthalate (DnOP), diisobutyl phthalate (DiBP), dimethyl phthalate (DMP), diethyl phthalate (DEP), benzyl butyl phthalate (BBP), bis(2-methoxyethyl) phthalate (DMEP), bis(4-methyl-2-pentyl) phthalate (BMPP), bis(2-ethoxyethyl) phthalate (DEEP), dipentyl phthalate (DPP), dihexyl phthalate (DnHP), bis(2-n-butoxyethyl) phthalate (DBEP), dicyclohexyl phthalate (DCHP), and dinonyl phthalate (DNP) were purchased from AccuStandard (New Haven, CT, USA). These selected 15 ingredients cover the major PAEs used in industrial processes, especially those applied as plasticizers. The methanol, formic acid, acetonitrile, hexane, and dichloromethane used were HPLC-grade. Silica gel, neutral alumina, and anhydrous sodium sulfate were activated in advance.

### 2.3. Sample Extraction and Purification

The plant and soil extraction procedures have been described in detail by Wei et al. (2020) [25] and Wang et al. (2015a) [19]. Each soil sample was weighed, amounting to 6 g, and placed into a 30 mL glass centrifuge tube. The extractant was a hexane: dichloromethane (1:1; *v*/*v*) mixture. Subsequently, 20 mL of the extractant mixture was added to the glass centrifuge tube with the soil sample, and shaken well. The sample was placed in the ultrasonic extraction system: the time was set to 20 min. After removing the tube, water was wiped off with absorbent paper, and then the tube was transferred to the centrifuge: the centrifugation setting was 2000 r/min, 10 min. The supernatant was transferred to the chicken heart bottle at the end of centrifugation. The above ultrasonic extraction and centrifugation operation was repeated three times. The flask containing the supernatant was nitrogen blown to near dryness, and the volume was fixed by adding hexane to the flask to a total volume of 3 mL, and shaken well. A glass syringe was used to transfer 1.5 mL of the fixation solution through a 0.22-μm glass fiber membrane to a chromatography vial, which was sealed and stored for detection. The detailed steps of plant sample extraction are shown in Text S1.

Total organic carbon (TOC) and pH assays are shown in Text S2.

### 2.4. Instrumental Analysis

PAEs were analyzed independently by GC/MS coupled with DSQII mass spectrometry (Thermo Fisher Scientific, Inc., Waltham, MA, USA). The extract was injected into a DB-5MS fused silica capillary column (30 m, 0.25 mm i.d., 0.25 μm film thickness, Agilent, Santa Clara, CA, USA) in splitless mode. Quantification was performed in electron ionization (EI) mode using a selected ion monitor (SIM). The GC conditions were as follows: the carrier gas was helium (>99.999% purity) at a flow rate of 1.2 mL/min; the injection volume was 1.0 μL; the syringe temperature was set to 250 °C; and the oven program was specified at 50 °C (maintained for 1 min), increased by 15 °C/min to 200 °C (maintained for 1 min), and then increased by 8 °C/min to 280 °C (maintained for 3 min). The post-treatment procedure was performed at 285 °C for 10 min. The GC/MS transmission line was set to 280 °C.

### 2.5. Quality Assurance and Quality Control

To avoid the influence of PAEs on the environment, no plastic products were used in the experiments; only glass products were used. To ensure the accuracy of the experimental procedure, and to determine whether there was any contamination, solvent blanks, procedure blanks, and duplicate samples were performed for some samples, according to the standard operating procedures. The target compounds were quantified using a five-point calibration curve (R^2^ > 0.999). The recovery rates of 15 PAEs in the samples ranged from 72.2 to 106.1%, and the limits of detection (LODs), calculated as signal-to-noise ratios of 3, were 0.00005–0.00028 mg/kg for PAEs. The glass was rinsed with ultrapure water and organic solvents, such as methanol, and placed in a muffle furnace for 5 h at 450 °C.

### 2.6. Statistical Analysis

Statistical analyses were performed using SPSS 22.0 (IBM, Armonk, NY, USA), Microsoft Excel 2021, and Origin 9 (OriginLab Inc., Northampton, MA, USA). Pearson’s correlation analysis was used to evaluate the relationships between contaminant concentrations in soil and plants, total organic carbon (TOC) in soil, and soil pH. ArcGIS 10.2 was used to map the spatial distribution of Σ15PAEs in soil and plants in coastal areas of South China.

### 2.7. Health Risk Assessments

Among the individual PAE congeners studied, DMP, DEP, DnBP, and DnOP were considered to be noncancerous compounds of human health relevance, whereas DEHP and BBP were carcinogens [19,36]. The main routes of PAE intake by residents in coastal areas of South China are dietary and nondietary. The dietary intake route represents food intake, including the intake of vegetables. Nondietary routes include soil ingestion, inhalation, and dermal exposure [37,38]. The noncarcinogenic and carcinogenic risk assessments related to exposure to PAEs were evaluated by the models recommended by the USEPA (2013) [36]. In the present study, *ADD* (mg/kg/day) is the average daily dose via nondietary routes, including soil ingestion (*ADD_ingest_*), inhalation (*ADD_inhale_*), and dermal contact (*ADD_dermal_*), as well as dietary routes (*ADD_intake_*), calculated according to Equations (1)–(4), respectively. The calculation of the hazard quotient (*HQ*) is presented in Equation (5). The noncancer risks of pollutants via nondietary and dietary pathways are presented as *HI*, and calculated via Equation (6). The carcinogenic risk (*CR*) of each pollutant was calculated using Equation (7).

(1) $$ADD_{ingest}=\frac{C_{soil}\times IRS\times EF\times ED}{BW\times AT}\times CF$$

(2) $$ADD_{inhale}=\frac{C_{soil}\times IhR\times EF\times ED}{PEF\times BW\times AT}$$

(3) $$ADD_{dermal}=\frac{C_{soil}\times SA\times AF\times ABS\times EF\times ED}{BW\times AT}\times CF$$

(4) $$ADD_{intake}=\frac{C_{plant}\times IRF\times EF\times ED}{BW\times AT}\times CF$$

(5) $$HQ_{i}=\frac{ADD_{i}}{RfD_{i}}$$

(6) $$HI=\sum^{\;}HQ_{i}$$

(7) $$CR=\sum^{\;}\left(ADD_{i}\times CFS\right)\;$$

The parameters used for calculating the noncarcinogenic and carcinogenic risks are shown in Text S3.

PAEs are known as endocrine-disrupting chemicals; even if the estrogenic effects of DEP, DnBP, BBP, and DEHP are very weak, they may pose a potential risk to human health. In the present study, to assess the health risks associated with potential estrogenic activity in plants, estrogenic equivalents (*EEQ*) were calculated using Equation (8):

(8) $$EEQ=\sum^{\;}(EP\;\times \;C_{\mathrm{plant}})\;$$

*EP* is the corresponding estrogenic activity; *C_plant_* (mg/kg) is the concentration of target phthalate in the plant. In the risk assessment related to estrogenic activity, 17β-estradiol (E_2_) was chosen as the standard estrogenic chemical to characterize the *EP* of each chemical, and the *EP* of E_2_ was set as 1. When estrogenic activity is weaker than E_2_, its *EP* is less than 1; otherwise, it is greater than 1. The unit of *EEQ* is usually expressed as ng E_2_/kg [39,40].

The value of parameters for human risk assessment and estrogenic equivalents are listed in Tables S2 and S3.

## 3. Results and Discussion

### 3.1. Overall Characteristics of PAE Contamination in Soil

Based on the analysis of 37 topsoil samples, the descriptive statistics of PAE monomer and total PAE concentrations in the topsoil of agricultural fields in the coastal areas of South China are shown in Table 1. PAEs were detected in all samples, indicating their ubiquity as environmental contaminants. The total concentrations of the 15 PAEs in soils were 0.445–4.473 mg/kg, with a mean value of 1.582 mg/kg, and the detection frequency was 100%. The Σ6 PAE concentrations, i.e., DEHP, BBP, DEP, DMP, DnBP, and DnOP, listed as priority pollutants by the UAEPA, ranged from 0.239–3.495 mg/kg, and the mean value was 1.329 mg/kg, with a detection frequency of 100%. Among the 15 PAEs, the detection frequencies of DEHP, DnBP, DiBP, DEP, and DMP were all 100%, and the detection frequencies of BMPP, DMEP, DEEP, DnHP, DnOP, DBEP, DNP, DPP, BBP, and DCHP were 89.0%, 86.0%, 86.0%, 78.0%, 76.0%, 73.0%, 70.0%, 70.0%, 59.0%, and 41.0%, respectively. The relative contributions of each PAE monomer in the soil are shown in Figure S1. Among all soil samples, DEHP had the highest concentration in the study area of coastal soils in South China, with a contribution of 24.47–90.27%, and an average contribution of 57.65%. In addition, DnBP and DiBP had contributions of 5.00–33.84% and 2.66–19.42%, and average contributions of 8.04% and 17.31%, respectively. This was followed by DMP and DEP, with contributions ranging from 0.08–20.08% and 0.026–15.19%, and mean contributions of 4.68% and 4.54%, respectively. The DEHP, DnBP, DiBP, DMP, and DEP accounted for 92.23% of the Σ15 PAE concentrations, indicating that they were the major contaminants of PAEs in coastal agricultural soils in South China. Due to the lack of data on soil PAE levels in past study areas, the temporal changes of PAEs in soils in the coastal areas of South China are inconclusive.

Some studies on soil PAEs in other regions and countries have been conducted, and are summarized in Table 2. The concentrations of PAEs detected in this study were comparable to those reported in some of these previous studies [16,35,37,41]. Ma et al. (2003) investigated greenhouse soils in Beijing, and found that the concentration of Σ6 PAEs ranged from 1.34 to 3.15 mg/kg [35]. Hu et al. (2003) conducted a soil survey in various regional cities in China, and found that the concentrations of Σ4 PAEs in four cities in northern China were 1.76–3.78 mg/kg, and the concentrations of Σ4 PAEs in two cities in northwestern China were 2.23–2.81 mg/kg [16]. Furthermore, the concentrations of Σ4 PAEs were 0.89–3.16 mg/kg in four cities in southern China, and the concentrations of Σ4 PAEs were 1.85–2.96 mg/kg in three cities in southwestern China. Yang et al. (2013) investigated agricultural soils in the Yangtze River Delta region, and found that the concentrations of Σ6 PAEs ranged from 0.716 to 3.251 mg/kg, and of Σ11 PAEs ranged from 0.794 to 3.461 mg/kg [41]. In recent years, Zhou et al. (2021) investigated agricultural soils in the Yellow Huaihai region, and found that the concentrations of Σ6 PAEs ranged from 0.239 to 3.495 mg/kg, and Σ16 PAEs ranged from 0.445 to 4.43 mg/kg [37]. Some studies have also reported higher concentrations of PAEs in some areas than those in this study [42,43,44]. Among them, Li et al. (2016) investigated the content of PAEs in vegetable plots covered with plastic film in the Shandong Peninsula in 2015, and the total content of Σ16 PAEs ranged from 1.374 to 18.810 mg/kg, with an average of 6.470 mg/kg [43]. These results indicate that the residual concentrations of PAEs in soils in different regions of China are in the same order of magnitude, with little variation between years and regions. The concentration levels in this study are in the middle range of the abovementioned studies, which may be related to the extensive survey area, and the more frequent agricultural activities in the coastal soils of South China. Moreover, the higher concentration may be related to the type of soil studied, such as mulched soil and greenhouse soil [19,43]. Since PAEs in plastics are easily released into the environment, plastic films containing high levels of PAEs may come into direct contact with soil through plastic or airborne deposition into soil, leading to increased PAE contamination in soil. Greenhouse vegetable soils and mulched soils have become priority areas for PAE contamination. In addition, farmers also discard large amounts of degraded-quality mulch in agricultural soil areas [45], which is likely to lead to additional accumulation of PAEs in the soil. Sediments and sewage sludge containing PAEs are also commonly used as fertilizers in agricultural activities, and are applied to agricultural fields in South China [45,46]. This can likewise lead to additional accumulation of PAEs in the soil.

For monomeric PAEs, DEHP and DnBP were the main residues of the 15 PAEs in coastal agricultural soils in southern China. The concentration range of DEHP was 0.129–2.628 mg/kg, with an average concentration of 0.947 mg/kg; the concentration range of DnBP was 0.073–1.109 mg/kg, with an average concentration of 0.259 mg/kg. DEHP and DnBP are deemed as two typical phthalates that are generally considered to be widespread in soils. Some studies had investigated these two PAEs in soils: Xu et al. (2008) investigated DEHP and DnBP in fluoridated soils in Harbin and Handan in 2006, and found that the concentration of DEHP in the black soil of Harbin was 0.44–4.20 mg/kg, and the concentration of DnBP was 2.75–14.62 mg/kg; DEHP concentration ranged from 1.15 to 7.99 mg/kg, and DnBP concentration ranged from 3.18 to 29.37 mg/kg in fluoride soil in Handan City [47]. These concentration ranges are significantly higher than in this study, probably due to more frequent agricultural activities, such as greenhouse cultivation in the north and more years of cultivation, resulting in higher levels of DEHP and DnBP residues in the soil. The DnBP content in this study was higher than that of DEHP, probably ascribing to differences in pollution sources and soil characteristics [47]. According to the commonly used US-recommended soil control criteria (Table S4), only DnBP exceeded the soil control criteria for the six priority contaminants, with an exceedance rate of 91.9%. However, the residual levels of all six PAEs were well below the soil treatment standard (Table S4). In addition to the six EPA priority contaminants, DiBP, which is an isomer of DnBP, also deserves attention in this study, as the average concentration of DiBP in coastal agricultural soils in southern China is second only to DnBP, and it also has a high detection frequency. Higher concentrations of DiBP have also been detected in soil studies from other regions of China [37,42]. The low concentrations of DiBP in foreign soils suggests that DiBP might be a specific PAE contaminant in China, which a few studies have reported, providing a new direction to the study of PAEs in the future.

### 3.2. Regional Differences in PAE Contamination

**Figure 1:**
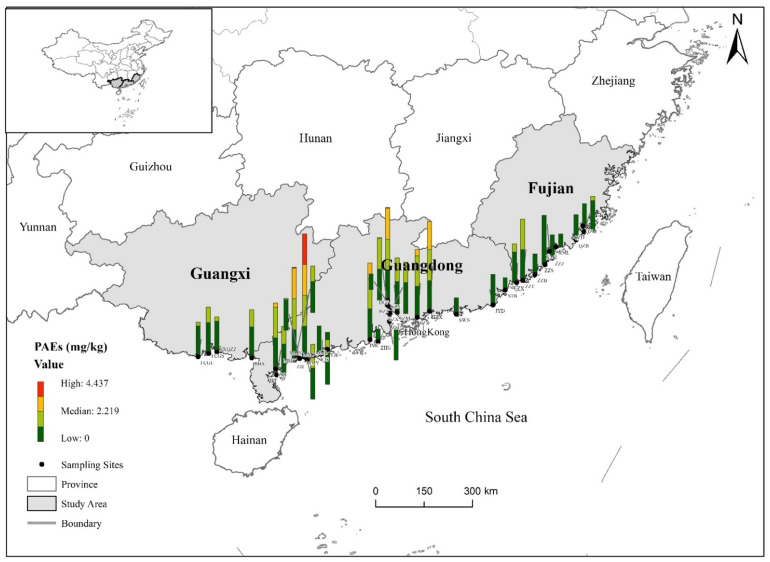
Concentration and geographical distribution of PAEs in agricultural soil in coastal areas of South China.

Since the source of PAEs is closely related to urban activities and agricultural cultivation patterns, the pollution characteristics of PAEs have some regional differences [15]. To visualize the concentration distribution, the spatial distribution of Σ15 PAE concentration is shown in Figure 1. Overall, the average concentration of Σ15 PAEs in agricultural soils in Guangdong Province was the highest, reaching 1.829 mg/kg, followed by Guangxi Province, reaching 1.478 mg/kg, and the lowest average level of Σ15 PAEs in agricultural soils was in Fujian Province, at 1.018 mg/kg. As shown in Figure 1, it can be seen that Σ15 PAE concentration is high at one location in Maoming City, which was mainly attributed to this location being surrounded by large agricultural fields with extensive cultivation, extensive mulch coverage, frequent agricultural activities, and high soil fertilizer application. Mulch is considered to be an important source of PAEs in agricultural soils, with DEHP, DiBP, and DBP being the main species detected in plastic films. The aging of plastic films, as well as evaporation of gases and leaching of water vapor from vegetable soils, can lead to higher levels of PAE contamination [43]. In addition, the data show that Σ15 PAE content in agricultural soil is high in the Pearl River Delta region of Guangdong Province. The main reason for this high level of contamination may be the rapid industrial development in the Pearl River Delta region, and thus the high release of PAEs from factories in the region. In addition, the high rate of urban development accompanied by agricultural and domestic waste also led to relatively high levels of PAEs in this region [48,54]. These results reveal a certain location dependence of soil contamination by PAEs, which can be attributed to regional differences in industrial product development, economic and agricultural activities, and soil type of specific geographical locations.

Regarding the monomeric PAEs, in general, the sampling sites did not show significant differences (Figure S1), with DEHP, DnBP, and DiBP being the major phthalate contaminants in all three South China coastal provinces; however, they did exhibit slightly different levels. In this study, DEHP and DnBP were generally higher than other monomeric PAEs, probably because DEHP and DnBP are the main plasticizers used in polyvinyl chloride (PVC) materials and other plastic products [55]. Among them, DEHP is much higher than DnBP, probably because, in soft PVC products, DEHP can be added up to 40%, while the concentration of PAEs in plastic products is 10–60% [56]. Additionally, the use of plastic products is essential during agricultural activities, such as mulching and greenhouse film, which are considered to be important sources of PAEs in agricultural soil in coastal areas of South China. In addition, PAE contamination is likely to be caused by agricultural nonpoint sources, including pollution from livestock, fertilizers, and pesticides [37]. DEHP and DnBP were detected in the most widely used fertilizers, with organic fertilizers containing five times higher concentrations of DnBP than other PAEs [45]. Moreover, among the PAE congeners, DEHP has a higher relative molecular mass and longer carbon chain length, resulting in low water solubility (S_W_) and a large octanol–water partition coefficient (K_OW_); therefore, it is less mobile in soil and easily absorbed by soil. The length and relative molecular weight of alkyl chains are the main factors affecting the degradation rate and corresponding half-life of PAEs [37]. However, PAEs with longer alkyl chains, including DEHP, have a slower biodegradation rate [15]; thus, DEHP is more likely to accumulate in the soil at high concentrations. DnBP is a low-molecular-weight PAE that has low durability due to its volatility and water extractability, and is easily released into the environment, resulting in a high accumulation of DnBP in soil. Other low-molecular-weight PAEs, including DiBP, DMP, and DEP, are also widely used in cosmetics and personal care products. DiBP and DnBP are also used in products such as cellulose, special adhesive formulations, and epoxy resins [57]. In contrast, DMP and DEP are used in smaller amounts, have lower soil sorption coefficients, and are less tightly bound to soil organic matter [37]; consequently, their soil residue levels are lower. Correlation analysis of the major monomeric PAEs, including DEHP, DnBP, DiBP, DMP, and DEP, in coastal agricultural soils of South China, showed that the concentrations of DnBP, DEHP, and DiBP were significantly correlated with each other (*p* < 0.01), and that DMP and DEP were significantly correlated with each other (*p* < 0.01). These significant correlations indicate that some PAEs may have similar sources and input pathways in agricultural soils.

TOC and pH are considered to be important factors affecting the behavior and occurrence of PAEs [17]. In the coastal agricultural soils of South China studied in this study, soil TOC content ranged from 0.41 to 36.01 g/kg, with a mean value of 10.47 g/kg. The pH measurements ranged from 5.13 to 8.85, with a mean value of 6.59 (Table S5). Pearson’s correlation matrix was considered significant at *p* < 0.01 and *p* < 0.05, with coefficients indicating that Σ15 PAE concentrations and soil TOC were significantly correlated with each other (*p* < 0.05), while Σ15 PAE concentration and soil pH were not significantly correlated. This suggests that lipophilic hydrophobic organic matter may be adsorbed to soil organic matter to varying degrees, depending on their physical properties and direct interactions.

### 3.3. Concentration and Distribution of PAEs in Plants

Table 3 shows the descriptive statistics of PAE monomers and total PAE concentrations in plants (leafy vegetables) at 37 sampling sites, corresponding to soils in coastal areas of South China. The total concentrations of 15 PAEs in plants ranged from 2.176 to 30.278 mg/kg, with a mean value of 8.712 mg/kg and a detection frequency of 100%. The total concentrations of six PAEs ranged from 1.329 to 28.685 mg/kg, with a mean value of 7.087 mg/kg and a detection frequency of 100%. As in the case of agricultural soils, the detection frequencies of DEHP, DnBP, DiBP, DEP, and DMP were all 100% among the 15 PAEs. The detection frequencies of DCHP, DMEP, DNP, DEEP, DnHP, DnOP, BBP, BMPP, DBEP, and DPP were 86.0%, 78.0%, 51.0%, 43.0%, 35.0%, 22.0%, 16.0%, 16.0%, 5.0%, and 3.0%, respectively. Figure S2 shows the relative contribution of each PAE monomer in plants at each sampling site on the South China coast. Among all the plant samples, DEHP had the highest concentration in the plants of the study area, with a contribution of 24.43–80.56% and a mean contribution of 47.59%. Following this, DnBP and DiBP contributed 0.95–58.96% and 4.27–38.80%, respectively, with corresponding mean contributions of 23.05% and 12.87%. The next in line were DMP and DEP, with contribution rates of 0.06–18.53% and 0.07–13.65%, and average contribution rates of 5.40% and 3.80%, respectively. DEHP, DnBP, DiBP, DMP, and DEP accounted for 92.71% of the concentration of Σ15 PAEs, indicating that they were the main contaminant of PAEs in coastal plants in South China. The average concentrations of the six priority control compounds in plants were in the order of DEHP > DnBP > DMP > DEP > BBP > DnOP in agricultural soils. Statistical analysis showed that there was no linear correlation between the levels of Σ15 PAEs in soil and plants from the same sampling site. The accumulation of PAEs in plants is significantly higher compared to PAEs in agricultural soils. This is probably because the plants can absorb PAEs not only from environmental substrates, such as soil and water, through their roots, but also from the air through their leaves [58]. The volatilization and atmospheric deposition of individual PAEs in construction materials and plastic products are important reasons for the increase in pollutant concentrations [15]. PAEs can also accumulate in plants via the leaves through pesticide spraying and fertilization [59].

Some studies on PAEs in plants have been carried out in other regions or countries [19,25,27,51,54,60,61]. Mo et al. (2009) reported that six PAEs were found in 11 vegetable species, collected from nine farms in the Pearl River Delta of southern China in 2007, with concentrations of Σ6 PAEs ranging from 0.15 to 11.2 mg/kg [54].

Overall, compared to results obtained from other study regions, the concentrations of Σ6 PAEs in plants from coastal areas in southern China were relatively high (Table 4). The main reason for this is probably the different levels of PAE contamination in plants in different regions and at different times, including the differences in the bioconcentration capacity of PAEs in plants due to different plant species.

In the present study, DEHP and DnBP accumulated in plants at higher concentrations than other monomeric PAEs. The concentrations ranging from 0.696 to 11.080 mg/kg with a mean of 4.114 mg/kg for DEHP, and 0.028–16.273 mg/kg for DnBP, with a mean concentration of 2.282 mg/kg. Concentrations in coastal plants in South China were significantly higher than in Netherlands vegetables (geometric mean of DEHP concentration was 0.0418 mg/kg, and DnBP concentration was below the detection limit) [51], but lower than those in *Benincasa hispida* collected in southern and northern China, with fresh weight concentrations ranging from 2.6 to 75.5 mg/kg [60]. Overall, the monomeric PAEs in coastal plants in South China were consistent with those in the soil. The average concentration of DiBP in coastal plants in South China was only second to DnBP and DEHP in soil, ranging from 0.110 to 7.916 mg/kg, with an average concentration of 1.139 mg/kg.

Chen et al. (2017) investigated the DiBP content in the leaves of greenhouse vegetables in 10 cities in China. Their results indicate that the mean concentration of DiBP was lower than that of DEHP and DnBP, but was significantly higher than that of DMP and DEP, which was consistent with the present study [61]. The lower concentrations of other monomeric PAEs were due to their low-level addition to agrochemical and plastic wastes, resulting in lower accumulation in the soil, and limited uptake by plant roots due to differences in their physical properties, such as hydrophilicity. In addition, they are less abundant in other environmental matrices, such as air, which leads to lower accumulation in plants from various environmental matrices. The different degradation patterns of different PAE congeners in plants may also lead to their different distribution in plants [62].

**Table 4:** Comparison of concentration of PAEs in plants of different regions (mg/kg, dry weight).

Location	Plant Types	DMP	DEP	DnBP	BBP	DEHP	DnOP	Σ_6_ PAEs	Σ_15_ PAEs	References
Pearl River Delta	Vegetables	ND–0.69	ND–0.084	ND–2.03	ND–9.7	ND–9.3	ND–0.47	0.15–11.2	-	[54]
southern and northern provinces in China	*Benincasa hispida*	NA	NA	NA	NA	2.6–75.5	NA	-	-	[60]
Netherlands	Vegetation	NA	NA	ND	NA	0.0418b	NA	-	-	[51]
Dalian of Northeast China	Plant	NA	NA	1.33b	NA	2.84b	NA	2.44–21.8	-	[27]
Suburban plastic film greenhouses	Vegetables	ND–0.15	ND–0.35	0.13–1.81	ND–0.09	0.12–5.82	ND–1.31	0.51–7.16	-	[19]
Siyang	Leaves of greenhouse vegetables	0.457b FW	0.873b FW	2.42b FW	NA	1.68b FW	NA	6.12b FW	-	[61]
Shenyang		0.225b FW	0.235b FW	1.15b FW	NA	0.81b FW	NA	3.32b FW	-	[61]
Beijing		0.142b FW	0.118b FW	1.16b FW	NA	1.25b FW	NA	3.53b FW	-	[61]
Shouguang		0.159b FW	0.106b FW	1.01b FW	NA	1.31b FW	NA	2.95b FW	-	[61]
Xianyang		0.148b FW	0.140b FW	0.50b FW	NA	1.04b FW	NA	2.48b FW	-	[61]
Haimen		0.486b FW	0.282b FW	1.26b FW	NA	0.82b FW	NA	3.38b FW	-	[61]
Nanjing		0.394b FW	0.446b FW	1.92b FW	NA	1.03b FW	NA	4.81b FW	-	[61]
Changshu		0.182b FW	0.219b FW	2.10b FW	NA	1.41b FW	NA	4.91b FW	-	[61]
Fuzhou		0.193b FW	0.188b FW	0.76b FW	NA	1.14b FW	NA	3.36b FW	-	[61]
Kunming		0.156b FW	0.152b FW	0.80b FW	NA	1.24b FW	NA	2.90b FW	-	[61]
Yellow River Delta	Vegetables	ND–0.036	ND–0.063	ND–1.300	ND–0.034	0.002–15.700	ND–0.154	0.011–16.400	-	[25]
The coast of South China	Plant	0.004–1.196	0.098–1.003	0.028–16.273	ND–0.277	0.696–11.080	ND–0.046	1.329–28.684	2.175–30.275	This study

Note: ND, not detected; ‘‘-’’, not included in the study; NA, not available; ‘‘b’’ the value presented as the average value; ‘‘FW’’ the value presented as fresh weight.

**Figure 2:**
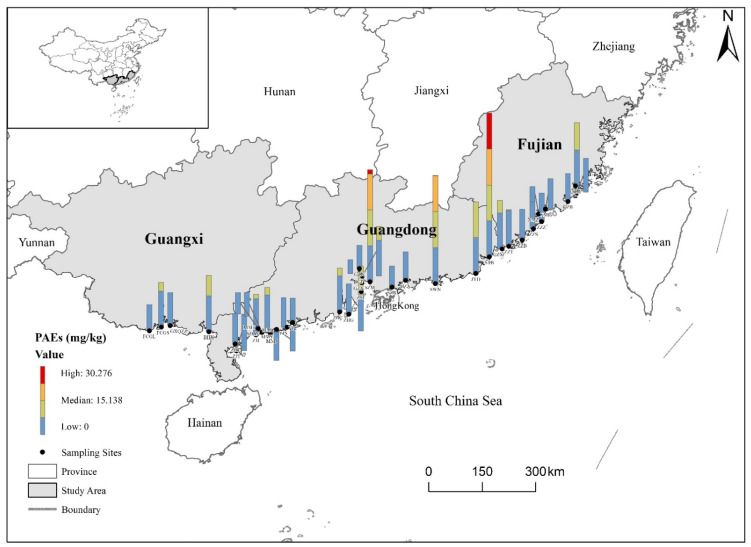
Concentration and geographical distribution of PAEs in plants in coastal areas of South China.

PAEs in plants mainly originate from soil and air deposition, and are closely related to agricultural activities and industrial production [15]. The contamination characteristics of PAEs in plants also have some regional variability. The spatial distribution of Σ15 PAEs in coastal plants of South China is shown in Figure 2. In general, similar to the distribution of Σ15 PAEs in coastal soils of South China, the average concentration of Σ15 PAEs in agricultural soils of Guangdong Province was the highest, reaching to 9.410 mg/kg, followed by Guangxi Province, reaching 8.424 mg/kg, while the average concentration of Σ15 PAEs in agricultural soils of Fujian Province was the lowest, at 6.977 mg/kg. As can be seen in Figure 2, there were three sampling sites in the coastal area of Guangdong Province with high levels of Σ15 PAEs, located in Shenzhen city, Shantou city, and Shanwei city. Although the sampling sites were located on agricultural soils, there are many factories in the surrounding areas, and the plants are easily affected by the “three wastes” of the city during the growth process. Frequent agricultural activities will lead to an increase in PAE residues in the soil, which will affect the concentration level of PAEs in plants. Correlation analysis of the major monomeric PAEs, including DEHP, DnBP, DiBP, DMP, and DEP, in coastal agricultural plants of South China, showed that the concentrations of DnBP, DEHP, DEP, and DiBP were significantly correlated with each other (*p* < 0.05), as were the concentrations of DnBP and DEP (*p* < 0.01), and DMP and DEP (*p* < 0.01). These significant correlation results are similar to the analysis of coastal agricultural soils of South China, and indicate that some PAEs may have similar sources and input pathways, and that soil is an important source of PAEs in plants. There was no significant difference (*p* > 0.05) between the total concentration of Σ15 PAEs in coastal soils and coastal plants of South China, most likely due to the complex sources of PAEs in plants, which can uptake PAEs not only from the soil, but also from other sources, such as the “three wastes” [63,64].

### 3.4. Ecological and Human Risk Assessments of PAEs in Coastal Areas of South China

**Figure 3:**
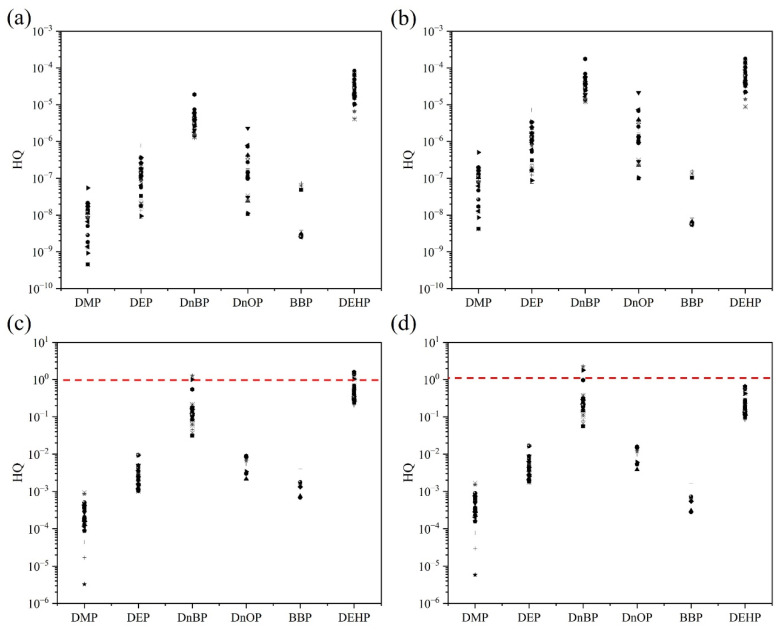
The hazard quotient (HQ) was calculated to assess the noncancer risk from phthalates (PAEs) in soil–plant systems. The dots represent the HQ of the noncarcinogenic risk of PAEs at each sampling site. Adult nondietary noncarcinogenic risk (**a**). Childhood nondietary noncancer risk (**b**). Adult dietary noncarcinogenic risk (**c**). Childhood dietary noncarcinogenic risk (**d**). The auxiliary line with an HQ value of 1.

The main pathways of human exposure to PAE contaminants include daily dietary intake, inhalation, and dermal contact [65]. The daily intake of PAEs by nonoccupational consumers is mainly through the dietary route, especially vegetables [19]. The plant samples investigated in this study mainly consisted of vegetables, so the concentrations of PAEs in plants in this study were representative. DEHP, BBP, DEP, DMP, DnBP, and DnOP are associated with noncancer risks, while DEHP and BBP are considered to be potentially carcinogenic. In this study, the ecological and human risk assessment of PAEs in soil–plant systems were assessed by dietary and nondietary pathways. The noncarcinogenic risks in the coastal area of South China are shown in Figure 3. For the nondietary and noncarcinogenic risks of the six PAEs in adults (Figure 3a) and children (Figure 3b), the hazard quotient (HQ) for all samples was less than 1. This result suggests that PAEs pose a limited noncarcinogenic risk to adults and children via nondietary routes, consistent with other studies [37]. Therefore, the nondietary pathway was not the main pathway that posed a risk to human health from PAEs. It was also found that the noncarcinogenic risk from nondietary routes was more severe in children than in adults in the coastal region of South China, suggesting that children should be more aware and alert to nondietary intake of PAEs. Of the six PAEs, BBP, DEP, DMP, and DnOP posed limited noncarcinogenic risk to adults (Figure 3c) and children (Figure 3d) via the dietary route. The HQ values were not greater than 1 for both adults and children. However, the mean adult HQs for DnBP and DEHP via the dietary route in the coastal region of South China were 0.178 and 0.597, respectively (Table S6), with 5.4% of plant samples having HQs greater than 1 for DnBP, and 21.6% of plant samples having HQs greater than 1 for DEHP. The mean HQs for children regarding DnBP and DEHP via the dietary pathway were 0.317 and 0.245, respectively (Table S7). Only 5.4% of the plant samples had an HQ greater than 1 for DnBP, and all samples had an HQ less than 1 for DEHP. This study has suggested that DEHP and DnBP pose a high noncarcinogenic risk via the consumption of coastal plants of South China. Therefore, it is important to evaluate the concentration and type of PAEs in the environmental matrix, and the dietary structure and habits of the population, to assess the human risk of PAEs. PAEs have been found to be significantly more sensitive in children than in adults, and as endocrine disruptors, PAEs hade a more serious impact on children’s development, which should be taken into account.

**Figure 4:**
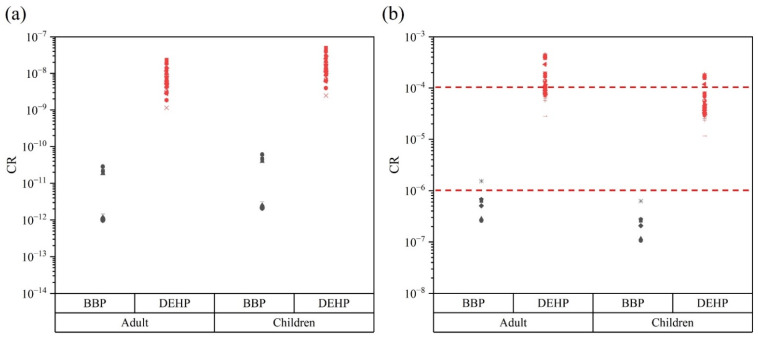
The carcinogenic risk values (CR) were calculated to assess the potential carcinogenic risk due to BBP and DEHP in soil–plant systems. The dots represent the CR values for the potential carcinogenic risk of BBP and DEHP at each sampling site. Nondietary carcinogenic risk for adults and children (**a**). Dietary carcinogenic risk for adults and children (**b**). The auxiliary lines with CR values of 10^−6^ and 10^−4^.

When CR < 10^−6^, 10^−6^ < CR < 10^−4^, 10^−4^ < CR < 10^−3^, 10^−3^ < CR < 10^−1^, and CR > 10^−1^ it can be considered as very low, low, medium, high, and very high carcinogenic risk. Figure 4a shows the outcome of the assessment of nondietary carcinogenic risk for adults and children at each sampling site in the coastal region of South China. It can be seen that children presented a higher nondietary carcinogenic risk than adults, probably due to immaturity and low-body-weight-induced susceptibility to toxicity. However, all samples had a CR < 10^−6^, hence the nondietary carcinogenic risk for adults and children was very low. Figure 4b shows the dietary carcinogenic risk assessment of adults and children at each sampling point in the coastal area of South China. It can be seen that, except for one sampling point, the CR of adults with BBP is between 10^−6^ < CR < 10^−4^, showing a low carcinogenic risk. The CR values of BBP of other samples were all less than 10^−6^, with a very low carcinogenic risk. The CR for DEHP at each sampling site represented low and medium carcinogenic risk. As shown in Tables S5 and S6, the mean CR values of BBP via the dietary pathway for adults and children were 1.05 × 10^−7^ and 4.30 × 10^−8^, respectively. The mean CR values for adults and children due to DEHP via the dietary pathway were 1.67 × 10^−4^ and 6.85 × 10^−5^, respectively. In general, unlike the nondietary pathway, adults exhibited a higher risk of carcinogenesis in the dietary pathway, which may be due to a longer duration of contaminant intake in adults than in children. Compared to other studies [19,25], the carcinogenic risk for adults and children in the coastal areas of South China was higher.

In the present study, the EEQ of plants in coastal agricultural soils of South China reached 98.75 ng E_2_/kg (Table S8), with the EEQ of DEP, DnBP, BBP, and DEHP being 0.15 ng E_2_/kg, 93.56 ng E_2_/kg, 3.8 ng E_2_/kg, and 1.23 ng E_2_/kg, respectively. The estrogenic effect of DnBP accounted for the largest proportion of this effect. In general, the PAE congeners that need to be focused on in terms of human risk are DnBP and DEHP, which pose greater ecological and human health risks. Overall, the health risks posed by PAEs through the dietary route should be monitored. Further study is needed to control the accumulation of PAEs from various environmental matrices into plants. PAEs can be biologically accumulated from plants to humans through the food chain, thus posing a serious health risk. Although the results indicate that the overall ecological and human health risks in soil–plant systems in the coastal areas of South China were low; however, the health and ecological risks of long-term low-dose exposures should not be ignored. In the further comprehensive assessment of PAEs, the potential risks to human health from exposure to PAE contaminants should be fully understood, and the different exposure pathways to PAEs need to be fully investigated.

The introduction of PAEs into ecosystems had raised concerns about health and food safety. In particular, our research found that the excessive residual of PAEs in soil can lead to their high accumulation in plants, which poses a worrying risk to human health. The potential disturbance of soil–plant systems could accelerate the transformation of PAEs and their metabolites from soil to vegetable. Particularly, the leafy vegetable samples presented in this study were all among the major crops grown in the coastal region of South China. The results of our study provide a reference for guiding vegetable production. Firstly, manufacturing industrial development may cause elevated levels of PAEs [25,66] due to the release of PAEs from these regions during production and disposal processes; thus, vegetable cultivation should be kept as far away as possible from these areas. Secondly, alternatives to plasticizers, such as vegetable-oil-based plasticizers, should be considered in the production of plastic products, especially plastic films. Nontoxic, biodegradable, and environmentally friendly plasticizers should also be developed. In addition, the use of plastic films in agricultural production activities such as mulching and greenhouse establishment should be reduced as much as possible. Biofertilizers containing PAEs-degrading bacteria can also be developed to promote the degradation of PAEs in the soil. The bioaccumulation of PAEs in crops can be reduced by selecting crop varieties that are low in PAE accumulation capacity [62]. Finally, there is a need to continuously monitor the pollution status of PAEs, and to adjust the restrictions in the industrial sector appropriately. We recommend that the Chinese government issue PAEs limit standards applied at the national context, including types and thresholds of PAEs in various matrices of the environment.

## 4. Conclusions

This study provided the contamination levels of 15 PAEs in agricultural soil–plant systems in coastal areas of South China. The levels of PAEs in the soil–plant system were higher than those reported in some other regions, suggesting that the rapid urban development and frequent agricultural activities in coastal areas of South China may lead to the accumulation of higher concentrations of PAEs in the environment. The levels of PAEs in the plants were significantly higher than those in the soil, which indicates that the plants were consistently accumulating higher levels of PAEs not only from the soil, but also from other environmental substrates (water, atmosphere). The ecological and human health risks of DnBP and DEHP are high, and PAEs in the soil–plant system are mainly from the plant pathway via daily dietary intake, and, to a lesser extent, from other pathways in the soil during daily life. High levels of PAEs are widely present under multiple pathways and the potential ecological and health risks that they pose to populations should be of greater concern and subject to regulatory control. In the context of agricultural safety and ecosystem health, future research should focus on reducing the exposure of individuals, especially children, to PAEs in dietary plants, including leafy vegetables. We recommend growing crop varieties with low PAE accumulation, and developing and using biofertilizers containing PAE-degrading bacteria to reduce the bioaccumulation of PAEs in crops.

## Figures and Tables

**Table 1:** Concentrations of Σ15 PAEs in agricultural soil samples in coastal areas of South China.

Compound	Detection Rate (%)	Mean (mg/kg)	Median (mg/kg)	Min (mg/kg)	Max (mg/kg)
**DEHP**	100	0.947	0.783	0.129	2.628
**DnBP**	100	0.259	0.228	0.073	1.109
**DiBP**	100	0.121	0.095	0.030	0.861
**DEP**	100	0.060	0.048	0.004	0.364
**DMP**	100	0.054	0.050	0.003	0.319
**BMPP**	89	0.006	0.002	ND ^a^	0.087
**DMEP**	86	0.007	0.0004	ND	0.042
**DEEP**	86	0.005	0.002	ND	0.016
**DnHP**	78	0.053	0.001	ND	1.449
**DnOP**	76	0.006	0.003	ND	0.054
**DBEP**	73	0.052	0.008	ND	0.753
**DNP**	70	0.003	0.002	ND	0.021
**DPP**	70	0.003	0.001	ND	0.010
**BBP**	59	0.004	0.001	ND	0.024
**DCHP**	41	0.005	ND	ND	0.072
**Σ6 PAEs**	100	1.329	1.182	0.239	3.495
**Σ15 PAEs**	100	1.582	1.272	0.445	4.437

^a^ ND, not detected.

**Table 2:** Comparison of concentration of PAEs in the soil of different regions (mg/kg, dry weight).

Location	Soil Types	DMP	DEP	DnBP	BBP	DEHP	DnOP	Σ_6_ PAEs	Σ_15_ PAEs	References
East China (7 cites)	Arable soils	ND	ND–1.29	0.21–1.38	NA	0.2–5.98	NA	1.34–7.14	-	[16]
Northeast China (3 cities)	Arable soils	ND	0.18–1.36	0.16–1.56	NA	0.51–2.15	NA	4.41–10.03	-	[16]
North China (4 cities)	Arable soils	ND–0.2	0.15–2.61	0.14–0.98	NA	0.51–2.18	NA	1.76–3.78	-	[16]
Northwest China (2 cities)	Arable soils	ND	0.18–0.25	0.38–0.39	NA	1.67–2.17	NA	2.23–2.81	-	[16]
South China (4 cities)	Arable soils	ND	ND–0.17	ND–0.26	NA	0.54–3.42	NA	0.89–3.16	-	[16]
Southwest China (3 cities)	Arable soils	ND	ND–0.37	0.51–0.64	NA	1.02–2.08	NA	1.85–2.96	-	[16]
Harbin	Black soils	NA	NA	2.75–14.62	NA	0.49–4.2	NA	-	-	[47]
Handan	Fluvo-aquic soils	NA	NA	3.18–29.37	NA	1.15–7.99	NA	-	-	[47]
Beijing	Greenhouse soils	0.01–0.02	0.01–0.05	0.34–1.66	NA	0.22–0.74	ND–0.09	1.34–3.15	-	[35]
Tianjin	Farmland soils	0.003–0.088	0.003–0.081	0.007–0.189	ND–1.79	0.039–2.37	ND–0.647	0.091–2.74	-	[48]
	Vegetable soils	0.002–0.101	0.002–0.114	0.013–0.285	ND–0.358	0.028–4.17	ND–9.78	0.050–10.4	-	[48]
	Orchard soils	0.003–0.032	0.003–0.03	0.02–0.138	ND–0.125	0.026–0.358	ND–0.728	0.053–1.08	-	[48]
	Wasteland soils	0.003–0.073	0.005–0.059	0.009–0.147	ND–0.471	0.051–0.494	ND–1.00	0.106–1.36	-	[48]
Sanjiang Plain	Cultivated topsoils	0.0266b	0.0349b	0.0285b	NA	0.0279b	NA	-	-	[49]
Yellow River Delta	Urban soils	0.002–0.060	ND–0.004	0.245–2.058	NA	1.465–6.320	ND–0.044	1.987–8.454	Σ11 PAEs: 2.096–8.527	[41]
	Suburb soils	0.001–0.065	0.001–11.24	0.166–1.450	NA	0.710–4.473	ND–0.142	1.007–16.007	1.079–19.504	[41]
	Rural soils	0.001–0.005	ND–0.001	0.136–1.039	NA	0.431–2.449	ND–0.068	0.716–3.251	0.794–3.461	[41]
Nanjing	Vegetable soils	ND–0.012	ND–0.007	ND–0.046	ND–0.002	0.204–0.704	0.002–0.019	0.314–0.564	-	[50]
	Using plastic soils	ND–0.016	ND–0.027	ND–1.41	ND–0.041	0.034–7.033	ND–1.739	0.480–9.676	-	[50]
Yellow River Delta	Agriculture soils	0.002–0.071	0.0005–0.0906	ND–1.5	ND–0.0122	ND–9.91	ND–0.274	0.068–9.33	Σ15PAEs: 0.167–9.37	[17]
Yellow River Delta	Agriculture soils	ND–0.002	ND–0.004	ND–0.069	ND–0.096	0.004–1.510	ND–0.075	0.005–1.580	-	[25]
Shandong Peninsula	Agriculture soils	ND–1.179	0.010–1.900	ND–9.855	ND–4.786	ND–2.943	ND–5.873	-	Σ16 PAEs: 1.374–18.810	[43]
Yinchuan	Agriculture soils	0.089–4.684	0.004–6.017	0.018–2.653	ND–0.039	0.069–2.693	ND–1.354	0.374–11.659	Σ16 PAEs: 0.391–11.924	[42]
Netherland	Soils	NA	NA	0.006b	NA	0.0318b	NA	0.038b	-	[51]
Scotland	Surface soils	NA	NA	NA	NA	0.025–1.60	NA	-	-	[52]
Serbia	Surface soils	0.006–0.038	0.005–0.011	0.030–0.145	0.001–0.013	0.13–2.04	0.0004–0.013	0.19–2.12	-	[53]
Xianyang	Vegetable soils	0.0213–0.0823	ND–0.067	0.037–6.313	ND–0.222	ND–3.871	ND–0.763	0.129–10.288	-	[44]
Huang-Huai-Hai	Agriculture soils	ND–0.116	ND–0.622	ND–1.417	ND–0.688	ND–2.314	ND–0.606	0.046–3.423	Σ16 PAEs: 0.0517–3.569	[37]
The coast of South China	Agriculture soils	0.003–0.319	0.004–0.364	0.073–1.109	ND–0.024	0.129–2.628	ND–0.054	0.239–3.495	0.445–4.437	This study

Note: ND, not detected; ‘‘-’’, not included in study; NA, not available; ‘‘b’’ the value presented as the average value.

**Table 3:** Concentrations of Σ15 PAEs in plant samples in coastal areas of South China.

Compound	Detection Rate (%)	Mean (mg/kg)	Median (mg/kg)	Min (mg/kg)	Max (mg/kg)
**DEHP**	100	4.114	2.896	0.696	11.081
**DnBP**	100	2.282	1.544	0.028	16.273
**DiBP**	100	1.139	0.778	0.110	7.916
**DEP**	100	0.292	0.214	0.098	1.003
**DMP**	100	0.374	0.340	0.004	1.196
**BMPP**	86	0.130	0.080	ND ^a^	0.737
**DMEP**	78	0.121	0.144	ND	0.286
**DEEP**	51	0.082	0.011	ND	0.690
**DnHP**	43	0.114	ND	ND	1.917
**DnOP**	35	0.005	ND	ND	0.046
**DBEP**	22	0.006	ND	ND	0.046
**DNP**	16	0.019	ND	ND	0.277
**DPP**	16	0.014	ND	ND	0.201
**BBP**	5	0.004	ND	ND	0.090
**DCHP**	3	0.004	ND	ND	0.158
**Σ6 PAEs**	100	7.087	4.985	1.329	28.685
**Σ15 PAEs**	100	8.712	6.505	2.176	30.276

^a^ ND, not detected.

## Supplementary Materials

The following supporting information can be downloaded at: https://www.mdpi.com/article/10.3390/ijerph19159516/s1. Text S1. The plant extraction procedures; Text S2. TOC and pH detection methods; Text S3. The parameters used for calculating the noncarcinogenic and carcinogenic risks; Table S1. Details of the 37 sampling sites; Table S2. Parameters for adults and children in the exposure risk assessment; Table S3. Parameters associated with different pollutants in the exposure risk assessment; Table S4. Control and treatment standards of PAEs in soil recommended by the Environmental Protection Agency in United States (mg/kg); Table S5. Soil TOC and pH concentrations in coastal South China; Table S6. Risk assessment of exposure to adults from PAE levels in soil–plant systems in coastal areas of South China; Table S7. Risk assessment of exposure to children from PAE levels in soil–plant systems in coastal areas of South China; Table S8. Potential estrogenic effect of PAEs via plant. Figure S1. Relative contributions of 15 PAEs in agricultural soil in coastal areas of South China; Figure S2. Relative contributions of 15 PAEs in plants in coastal areas of South China. References [39,67,68,69,70,71] are cited in the Supplementary Materials.
