# Supplementary material for: Characteristics and Health Risks of Phthalate Ester Contamination in Soil and Plants in Coastal Areas of South China
Source: Int J Environ Res Public Health. 2022 Aug 3;19(15):9516. doi: 10.3390/ijerph19159516 (PMC9367859; doi:10.3390/ijerph19159516)
Supplement: Supplementary file 1 [file ijerph-19-09516-s001.zip › ijerph-1823982-supplementary.pdf]

Supplementary Material

Characteristics and health risks of phthalate esters contamination in soil and plants in coastal areas of South China

Huanhuan Xing[a,b], Xiaolong Yu[b], Jiahui Huang[b], Xiaodong Du[a], Mengting Wang[a], Jianteng Sun[a,b,*], Guining Lu[a,*], Xueqin Tao[c]

[a] *Key Laboratory of Ministry of Education on Pollution Control and Ecosystem Restoration in Industry Clusters, Guangdong Provincial Engineering and Technology Research Center for Environmental Risk Prevention and Emergency Disposal, School of Environment and Energy, South China University of Technology, Guangzhou 510006, Guangdong, China*

[b] *Guangdong Provincial Key Laboratory of Petrochemical Pollution Processes and Control, School of Environmental Science and Engineering, Guangdong University of Petrochemical Technology, Maoming, Guangdong 525000, China*

[c] *College of Resources and Environment, Zhongkai University of Agriculture and Engineering, Guangzhou 510225, Guangdong, China*

**Corresponding author.*

E-mail address:

sunjianteng@zju.edu.cn *(J. Sun)*

GNLu@foxmail.com (G. Lu)

Contents

Text S1. The plant extraction procedures.

Text S2. TOC and pH detection methods.

Text S3. The parameters used for calculating the noncarcinogenic and carcinogenic risks.

Table S1. Details of the 37 sampling sites.

Table S2. Parameters for adults and children in the exposure risk assessment.

Table S3. Parameters associated with different pollutants in the exposure risk assessment.

Table S4. Control and treatment standards of PAEs in soil recommended by the Environmental Protection Agency in United States (mg/kg).

Table S5. Soil TOC and pH concentrations in coastal South China.

Table S6. Risk assessment of exposure to adults from PAEs levels in soil-plant systems in coastal areas of South China.

Table S7. Risk assessment of exposure to children from PAEs levels in soil-plant systems in coastal areas of South China.

Table S8. Potential estrogenic effect of PAEs via plant.

Figure S1. Relative contributions of 15 PAEs in agricultural soil in coastal areas of South China.

Figure S2. Relative contributions of 15 PAEs in plants in coastal areas of South China.

Text S1. Weigh 0.5 g of plant sample accurately, adding 20 ml of hexane: dichloromethane (1:1 v/v) extractant mixture to the glass centrifuge tube containing the plant sample. Then ultrasonic extraction for 20 min, centrifuge for 10 min. Then transferring the supernatant from the tube to another completely dry and clean chicken heart flask. The ultrasonic extraction and centrifugation were repeated three times. Before passing through the silica gel column (neutral alumina, neutral silica gel, and anhydrous Na_2SO_4 were filled into the glass column from bottom to top at 6 cm, 12 cm, and 2 cm), 5 ml of hexane and 15 ml of hexane: acetone (4:1; v/v) were measured and poured into the column to rinse the column. Then, the column was eluted with 45 mL acetone: hexane (1:4; v/v), and the eluate flowed into the cocktail vial. The resulting extracts were reduced nearly to dryness with a gentle nitrogen flow and diluted with hexane to 1.0 mL for instrumental analysis. And they were through the 0.22-μm glass fiber filter membrane before instrumental analysis.

Text S2. Soil pH was measured by a pH meter with a soil/water ratio of 1:2.5. Total organic carbon (TOC) was measured with an Elementar Vavio EL III elemental analyzer (Hanau, Germany).

Text S3. C_{soil} is the concentration of the pollutant in the soil (mg/kg); C_{plant} is the concentration of the pollutant in the plant (mg/kg); *IRS* is the soil ingestion rate (mg/day); *EF* is the exposure frequency (days/yr); *ED* is the exposure duration (years); *BW* is the body weight (kg); *AT* is the average lifetime exposure (days); *IhR* is the inhalation rate (m^3/day); *PEF* is the particulate emission factor (m^3/kg); *SA* is the dermal surface area (cm^2/day); *AF* is the soil adherence factor (mg/cm^2); *ABS* is the fraction absorbed dermally from the soil (unitless); *IRF* is the daily intake rate of the agricultural products (mg/day) by residents (*IRF* for children is set to 1/3 of that for adults). CF is the conversion factor (10^{-6} kg/mg). where *RfDi* (mg/kg/days) is the daily maximum permissible level of contaminants. ADD_i (mg/kg/days) is the average daily dosage, including dietary (plant intake, ADD_{intake}) and non-dietary (soil ingestion, dermal contact and inhalation, ADD_{ingest}, ADD_{dermal}, ADD_{inhale}); *CFS* (mg/kg/days) is the carcinogenic slope factor.

Table S1. Details of the 37 sampling sites.

Sampling location	East Longitude	Northern Latitude
CZX	117.100806	23.572944
FCGS	108.515856	21.599694
FCGL	108.212319	21.506575
MMT	110.951478	21.563847
MMN	111.049783	21.463131
MMJ	111.262819	21.423122
MML	111.419615	21.540649
MMY	111.184644	21.500889
QZD	118.979964	25.001342
QZM	118.950714	25.158578
QZB	118.760797	24.766167
XMZ	118.020906	24.450444
XML	118.193719	24.574706
YJS	111.681011	21.595306
YJL	111.822364	21.713825
ZJT	110.409367	21.004358
ZJB	110.375092	21.169489
ZJJ	110.897219	21.402325
ZZT	117.270911	23.635269
ZZB	117.610719	23.795119
ZZS	117.894083	24.079042
ZZZ	118.097258	24.257917
SWN	115.420097	22.694058
BHX	109.712433	21.481092

STB	116.780892	23.367864
JYD	116.439492	22.949333
GXQZZ	108.734553	21.637219
DGF	113.577308	22.88475
JMC	113.010436	21.981544
ZHG	113.237053	21.922653
ZSL	113.550114	22.491089
HZX	114.66896	22.77592
GZX	113.566014	22.718153
GZT	113.496656	22.942033
GZN	113.504078	23.07355
SZM	113.771397	22.741106
SZX	114.327478	22.607647

Table S2. Parameters for adults and children in the exposure risk assessment.

Parameter	Population	Value	Reference
IRS	Adults	100	[1]
	Children	200	
EF	Adults	350	[1]
	Children	350	
ED	Adults	26	[1]
	Children	6	
BW	Adults	80	[1]
	Children	15	
AT	Adults	9490 non-cancer)	[1]
		25550 (cancer)	
	Children	2190 (non-cancer)	
		25550 (cancer)	
IhR	Adults	13.25[a]	[2]
	Children	12[b]	
PEF	Adults	1.4×10^9	[1]
	Children	1.4×10^9	
SA	Adults	6032	[1]
	Children	2373	
AF	Adults	0.07	[1]
	Children	0.2	
IRF	Adults	652000	[3]
	Children	217000	

[a] The mean for male and female adults; [b] The mean for different ages of children.

Table S3. Parameters associated with different pollutants in the exposure risk assessment.

**Compound**	**ABS_a**	**CFS_a**	**RfD_i**	**EP_i**
BBP	0.1	1.9×10^{-3}	2.0×10^{-1}[4]	2.0×10^{-4}[5]
DEP	0.1	-	8.0×10^{-1}[6]	5.0×10^{-7}[5]
DEHP	0.1	1.4×10^{-2}	2.0×10^{-2}[4]	3.0×10^{-7}[5]
DnBP	0.1	-	1.0×10^{-1}[6]	4.1×10^{-5}[5]
DnOP	0.1	-	4.0×10^{-2}[6]	-
DMP	0.1	-	1.0×10^{1}[6]	-

[a] The parameters are recommend by the USEPA (2015)[1].

Table S4. Control and treatment standards of PAEs in soil recommended by the Environmental Protection Agency in United States (mg/kg).

**PAEs**	**control standards**	**treatment standards**
DMP	0.020	2.0
DEP	0.071	7.1
DBP	0.081	8.1
DEHP	4.350	50
BBP	1.125	50
DNOP	1.200	50

Table S5. Soil TOC and pH concentrations in coastal South China.

Soil Properties	Mean	Median	Min	Max
TOC	10.47[a]	9.14[a]	0.41[a]	36.01[a]
pH	6.59	6.34	5.13	8.85

[a] The unit of TOC is g kg^{-1}.

Table S6. Risk assessment of exposure to adults from PAEs levels in soil-plant systems in coastal areas of South China.

Congenger	HQ_{plant}	HQ_{ingest}	HQ_{inhale}	HQ_{dermal}
DMP	(2.92 ± 2.00) × 10^{-4}	(6.43 ± 7.14) × 10^{-9}	(6.08 ± 6.76) × 10^{-19}	(2.71 ± 3.01) × 10^{-9}
DEP	(2.85 ± 2.21) × 10^{-3}	(9.02 ± 9.86) × 10^{-8}	(8.53 ± 9.62) × 10^{-18}	(3.81 ± 4.16) × 10^{-8}
DnBP	(1.78 ± 2.49) × 10^{-1}	(3.10 ± 2.24) × 10^{-6}	(2.93 ± 2.09) × 10^{-16}	(13.1 ± 9.44) × 10^{-7}
DnOP	(1.10 ± 2.36) × 10^{-3}	(1.70 ± 2.89) × 10^{-7}	(1.61 ± 2.76) × 10^{-17}	(7.18 ± 12.2) × 10^{-8}
BBP	(2.76 ± 7.72) × 10^{-4}	(8.72 ± 16.4) × 10^{-9}	(8.26 ± 11.9) × 10^{-19}	(3.68 ± 6.91) × 10^{-9}
DEHP	(5.97 ± 4.14) × 10^{-1}	(2.11 ± 1.44) × 10^{-5}	(1.99 ± 1.34) × 10^{-15}	(8.90 ± 6.09) × 10^{-6}
	CR_{plant}	CR_{ingest}	CR_{inhale}	CR_{dermal}
BBP	(1.05 ± 2.97) × 10^{-7}	(3.32 ± 6.22) × 10^{-13}	(3.14 ± 5.89) × 10^{-22}	(1.40 ± 2.63) × 10^{-12}
DEHP	(1.67 ± 1.16) × 10^{-4}	(5.90 ± 4.04) × 10^{-9}	(5.58 ± 3.82) × 10^{-19}	(2.49 ± 1.70) × 10^{-9}

Table S7. Risk assessment of exposure to children from PAEs levels in soil-plant systems in coastal areas of South China.

Congenger	HQ_{plant}	HQ_{ingest}	HQ_{inhale}	HQ_{dermal}
DMP	(5.19 ± 3.55) × 10^{-4}	(6.86 ± 7.61) × 10^{-8}	(2.94 ± 3.26) × 10^{-18}	(1.63 ± 1.81) × 10^{-8}
DEP	(5.06 ± 3.92) × 10^{-2}	(9.62 ± 10.5) × 10^{-7}	(4.12 ± 4.51) × 10^{-17}	(2.28 ± 2.50) × 10^{-7}
DnBP	(3.17 ± 4.41) × 10^{-1}	(3.31 ± 2.38) × 10^{-5}	(1.42 ± 1.02) × 10^{-15}	(7.84 ± 5.66) × 10^{-6}
DnOP	(1.95 ± 4.19) × 10^{-3}	(1.81 ± 3.08) × 10^{-6}	(7.77 ± 13.2) × 10^{-17}	(4.30 ± 7.31) × 10^{-7}
BBP	(1.13 ± 3.16) × 10^{-4}	(2.15 ± 4.03) × 10^{-9}	(9.20 ± 1.73) × 10^{-19}	(5.10 ± 9.59) × 10^{-9}
DEHP	(2.45 ± 1.69) × 10^{-1}	(5.19 ± 3.55) × 10^{-5}	(2.22 ± 1.52) × 10^{-15}	(12.3 ± 8.42) × 10^{-6}
	CR_{plant}	CR_{ingest}	CR_{inhale}	CR_{dermal}
BBP	(4.30 ± 12.0) × 10^{-8}	(8.16 ± 15.3) × 10^{-12}	(3.50 ± 6.56) × 10^{-22}	(1.94 ± 3.63) × 10^{-12}
DEHP	(6.85 ± 4.75) × 10^{-5}	(14.5 ± 9.94) × 10^{-9}	(6.22 ± 4.26) × 10^{-19}	(3.45 ± 2.36) × 10^{-9}

Table S8. Potential estrogenic effect of PAEs via plant.

PAE compounds	Plant	
	**Mean value (mg/kg)**	**EEQ (ng E_2/kg)**
DEP	0.29 ± 0.23	0.15 ± 0.11
DnBP	2.28 ± 3.18	93.56 ± 130.40
BBP	0.02 ± 0.05	3.80 ± 10.64
DEHP	4.11 ± 2.85	1.23 ± 0.86
Total	6.70 ± 5.19	98.75 ± 130.64[a]

[a] The sum EEQ levels of DEP, DnBP, BBP, DEHP.

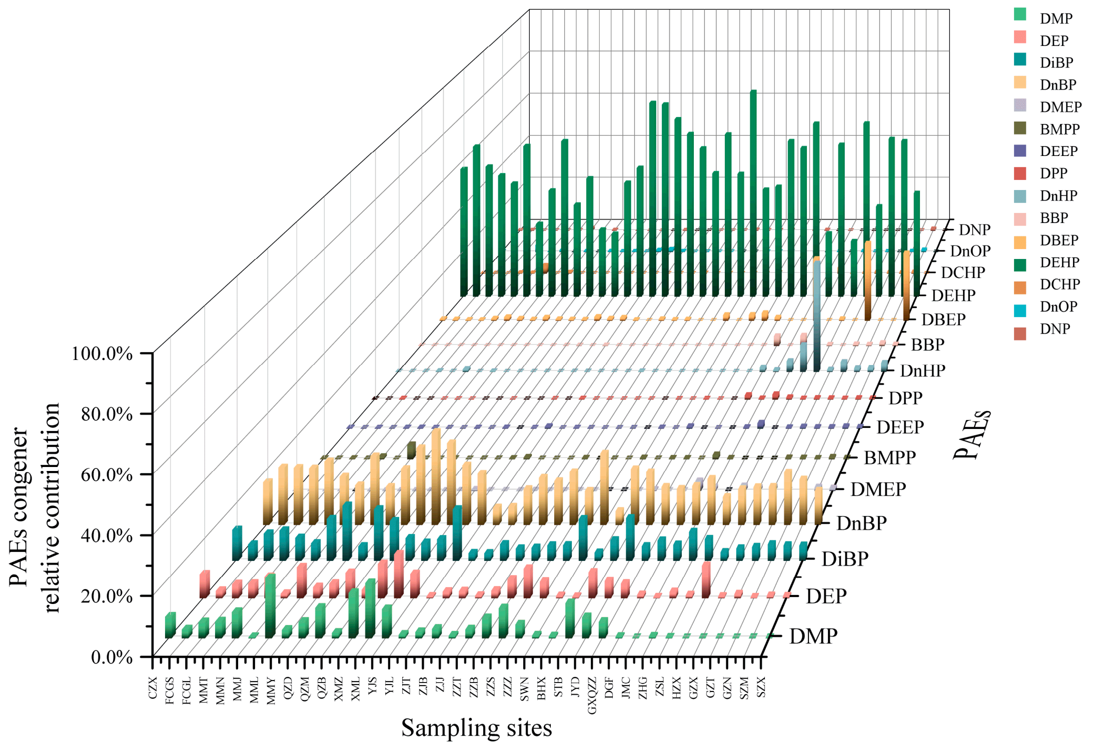

Figure S1. Relative contributions of 15 PAEs in agricultural soil in coastal areas of South China.

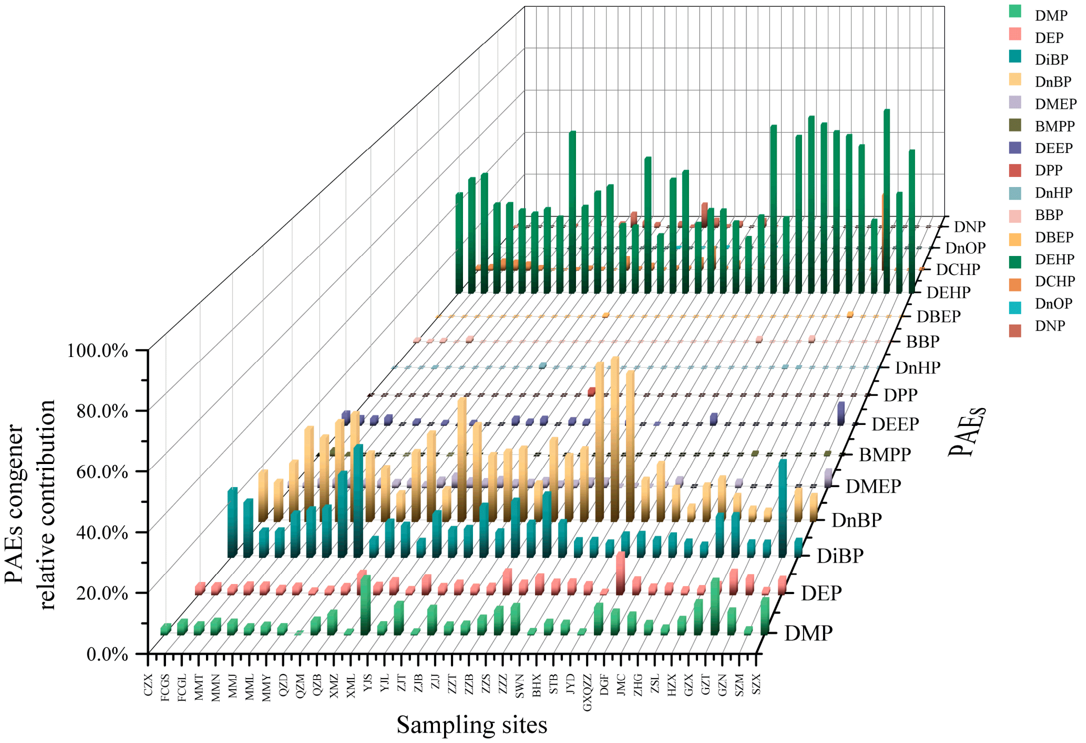

Figure S2. Relative contributions of 15 PAEs in plants along the coast of South China.

References

1. USEPA. *Mid-Atlantic RISK Assessment.* ; Untied States Environmental Protection Agency: Washington, DC, USA, 2015.
2. USEPA. *Exposure factors handbook, EPA/600/P-95/002F; Environmental Protection Agency*; Office of Research and Development: Washington, DC, 1997.
3. China, N.B.o.S.o. *China Statistical Yearbook*; China Statistic Press, Beijing., 2015.
4. Zeng, H.-H.; Zhang, H.-X.; Wu, X.; Gu, H.-X.; Zhang, L.-Z.; Liu, Y.-Y.; Zhao, X.-F.; Li, J.-H. Pollution levels and health risk assessment of particulate phthalic acid esters in arid urban areas. *Atmospheric. Pollut. Res*. **2017**, *8(1)*, 188−195.
5. Zeng, L.-J.; Huang, Y.-H.; Chen, X.-T.; Chen, X.-H.; Mo, C.-H.; Feng, Y.-X.; Lü, H.; Xiang, L.; Li, Y.-W.; Li, H.; Cai, Q.-Y.; Wong, M.-H. Prevalent phthalates in air-soil-vegetable systems of plastic greenhouses in a subtropical city and health risk assessments. *Sci. Total. Environ*. **2020**, *743*, 1−11.
6. Wang, L.; Liu, M.; Tao, W.; Zhang, W.; Wang, L.; Shi, X.; Lu, X.; Li, X. Pollution characteristics and health risk assessment of phthalate esters in urban soil in the typical semi-arid city of Xi'an, Northwest China. *Chemosphere*. **2018**, *191*, 467−476.
